# Promoting access of hydroxyurea to sickle cell disease individuals: Time to make it an essential medicine

**DOI:** 10.12688/f1000research.111300.1

**Published:** 2022-05-20

**Authors:** Manase Kilonzi, Hamu Mlyuka, Agnes Jonathan, Hilda Tutuba, Lulu Chirande, Paschal Rugajo, Irene Kida, Emmanuel Balandya, Julie Makani, Nathanael Sirili

**Affiliations:** 1 Muhimbili University of Health and Allied Sciences, Dar es Salaam, Tanzania

**Keywords:** Hydroxyurea, Quality of Life, Sickle Cell Disease and Tanzania

## Abstract

Hydroxyurea (HU) alone has the potential to prevent one out of every three deaths due to sickle cell disease (SCD) and almost all forms of disabilities caused by SCD. However, in Tanzania, only one out of every six registered SCD patients in the SPARCO-Tanzania Sickle Cell Cohort use HU. We conducted studies to understand factors influencing utilization of HU in Tanzania and discovered that among the reason for low utilization of HU include HU is classified as anticancer medication, only hematologists are supposed to prescribe HU, limited HU prescription to only National and Specialized hospitals, a special permit is required to access HU using National Health Insurance Fund (NHIF) scheme and limited importation and absence of local manufacturing of HU limit availability of this important drug in Tanzania. Therefore, with this brief, the government should allow prescription of HU to the district hospitals level, should allow all clinicians with a minimum of a Bachelor of Medicine to prescribe HU, and accessibility of HU through NHIF should be friendly.

## Introduction

Sickle cell disease (SCD) is an inheritable lifetime disease whereby red blood cells (RBC) (which are the vehicles for transportation and distribution of oxygen in the body) change shape and appear as a sickle. The sickled RBCs fail to pass smoothly in small blood vessels hence they accumulate and cause occlusion. ^1^ The blockage of the blood vessels results in poor blood supply, episodes of severe pain, and damage of affected parts of the body, particularly the brain, kidney, spleen, lungs, bones, and heart. This reduces the quality of life, and when unattended, results in 50-90% risk of deaths, ^2^ brings social and economic burden to the affected one, family and the nation at large. ^3^

Tanzania has the fourth highest burden of SCD in the world. Every year more than 11,000 children are born with SCD in Tanzania. ^2^ In the absence of care, the majority of children with SCD will not live to adulthood. In Tanzania, SCD contributes to approximately 7% of all deaths among children below five years of age. ^4^ In addition, the mean lifespan of Tanzanians with SCD is 33 years ^4^ which is half the average lifespan of the general population.

Available interventions to improve the quality of life of the individuals with SCD include awareness creation, newborn screening, preventive treatment and vaccines against bacterial infections, daily Vitamin B9 supplement, malaria prevention, and medications, routine blood transfusion, bone marrow transplant (a cure but expensive and not readily available), correction of the defective gene (a cure but expensive and not readily available) and use of hydroxyurea (HU). Of all the interventions, HU has proved to be cost-effective and safe. Currently, HU is the only medication used by those with SCD and its benefit have outweighed the risk. The uses of HU among SCD individuals have the following benefits: prevention of brain damage by stroke, prevention of renal failure, liver failure, and infections. Furthermore, HU prevents malaria infection, reduces the frequency of blood transfusion and the risk of death.

### Utilization of HU among SCD individuals in Tanzania

Despite HU being a simple oral medication with more than 30 years of evidence of being very effective and safe, it is not readily available, affordable, and accessible to patients with SCD in Tanzania. ^5^ So far, out of 5,064 registered SCD patients at SPARCO-Tanzania Sickle Cell Cohort, only 15.68% (794) receive HU.

## Methods

This policy brief has been prepared after conducting two qualitative pieces of research and managed to establish reasons for the underutilization of HU among SCD individuals in Tanzania. ^6^^,^^7^ Additionally, we conducted a literature review to gather information with regards to SCD and the uses of HU. One five year follow-up study involving 1,700 participants established reasons for mortality among patients with SCD in Tanzania. ^3^ Another three year randomized controlled clinical trial involving 600 participants established the effectiveness, safety, and feasibility of HU among patients with SCD in East and Central Africa. ^8^ Another study followed up 299 patients with SCD for 17.5 years to establish the long-term risks and benefits of HU. ^9^ Other sources of data came from two scoping reviews articles from Tanzania, ^1^^,^^2^ Tanzania National Treatment Guideline and Essential Medicines List, ^10^^,^^11^ Health Sector Strategic Plan 2021-2026 ^12^ and Strategic and Action Plan for Prevention and Control of Non-Communicable Diseases in Tanzania 2016-2020. ^13^

### Ethical approval

The study was conducted according to the guidelines of the Declaration of Helsinki, and approved by the Institutional Review Board (or Ethics Com-mittee) of MUHAS (Ref. No. DA. 282/298/01.C/. and 31/07/2020).

### Consent statement

Written informed consent was obtained from all subjects involved in the study.

**Figure 1.**
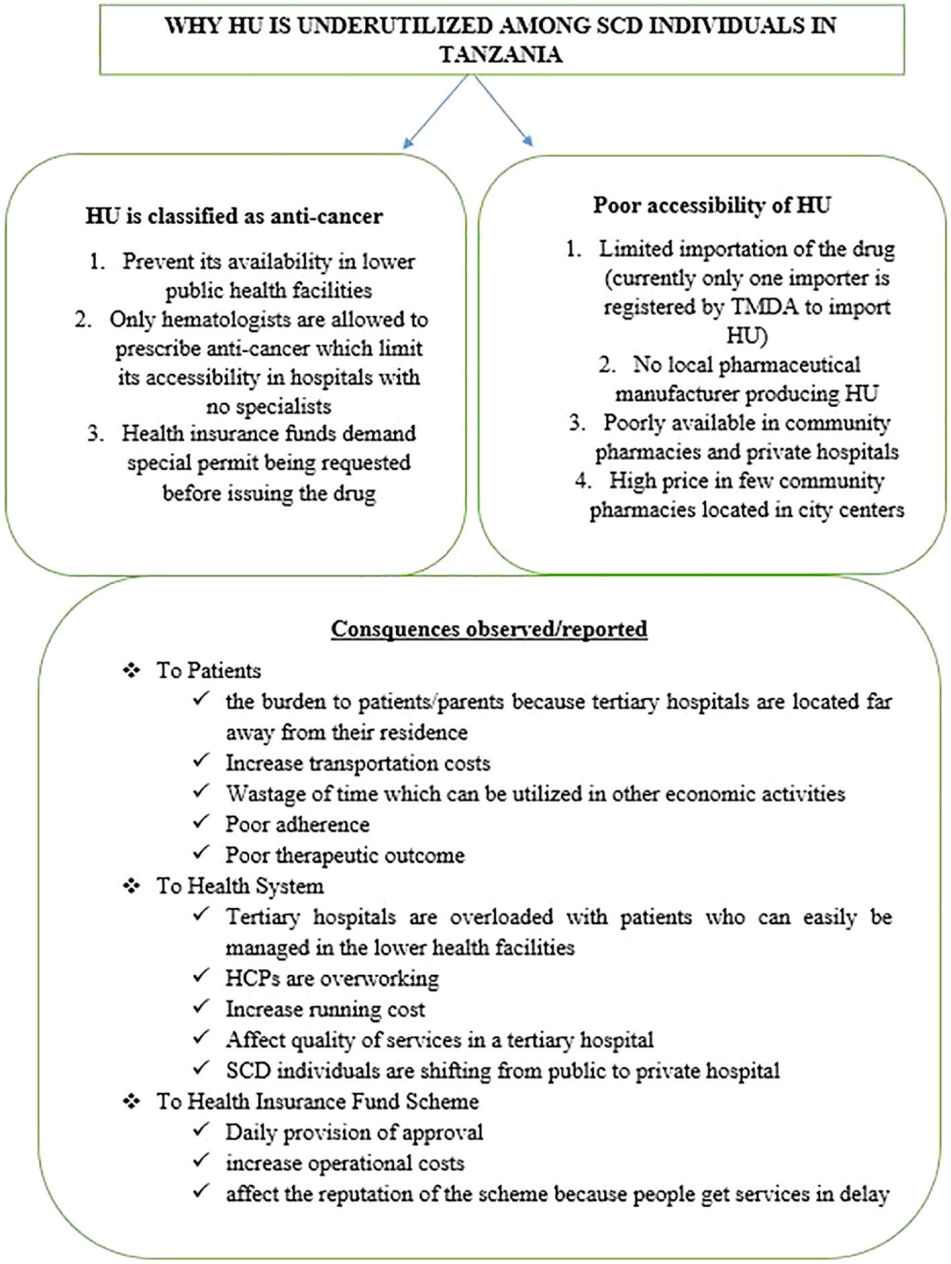
Summarize reasons for underutilization of HU among SCD individuals and its consequences.

**Policy gaps to be addressed ( Figure 1)**

**What should be done to improve utilization of HU among SCD individuals in Tanzania**

To achieve the goal of ensuring 70% of SCD patients receive standardized care and treatment and reduction of 50% of SCD-related deaths as stated in the strategic and action plan for the prevention and control of non-communicable diseases in Tanzania 2016 – 2020 ( *section 3.9 Expected Outcomes*), ^13^ the Ministry of Health should consider:

1. Extending prescription of HU to patients with SCD attending regional and district hospitals which have laboratory facilities for monitoring of blood parameters for patients on treatment.
2. Re-categorizing HU as the medication for cancer and non-cancer diseases.
3. Discussing with NHIF and remove the need for a special permit when issuing HU to SCD individuals.
4. Providing HU to individuals with SCD under a vertical program.
  • Capitalize on the experiences from tuberculosis, HIV/AIDS, and Neglected Tropical Diseases control programs.
5. Including HU in the subsidization scheme as an additional incentive on top of its inclusion in the Tanzania Orphan Drug Regulation of 2018.
  • Capitalize on the experiences from antimalarial medications, particularly Artemether-Lumefantrine.
6. Providing a more supportive environment (in collaboration with Ministries responsible for Finance, Industry, and Business) to local pharmaceutical manufacturers in terms of more subsidization of raw materials and infrastructures for manufacturing of HU for SCD in Tanzania.
  • This will help to realize priory number VIII in the health sector strategic plan 2021-2026 ^12^ which aims at “ *Improvement of research and development in health services to establish and strengthen research mechanisms on domestic pharmaceutical manufacturing that meet international standards for domestic and export use*”.
7. Creating (in collaboration with health research institutions) an easily accessible platform of reliable data on the burden of SCD and the need for HU in Tanzania which will help local pharmaceutical importers and manufacturers during the establishment of estimated demand and application for registration of HU.

## Data availability

Data availability statement: Data are not available publicly because they contain sensitive inter-view information and participants did not consent for their interviews to be shared publicly. The data are available from The Directorate of Research and Publication Muhimbili University of Health and Allied Sciences (contact via drp@muhas.ac.tz Tel.: +2552150302-6) for researchers who will be able to explain the reasons why they want access to the confidential information. Furthermore, the researcher should be affiliated to the registered institution.

## Authors contribution

MK, HJM and NS prepared the first draft of the policy brief. AJ, LC, IMK, HT, PR, EB and JM reviewed and improved the policy brief. All authors have read and agreed to the submitted policy brief draft.
