# Supplementary material for: Promoting access of hydroxyurea to sickle cell disease individuals: Time to make it an essential medicine
Source: F1000Res. 2022 May 20;11:554. [Version 1] doi: 10.12688/f1000research.111300.1 (PMC10457563; doi:10.12688/f1000research.111300.1)
Supplement: Peer review history

# Peer review: Reviewer response for version 1

**DOI:** 10.5256/f1000research.123006.r193800

**Published:** 2023-08-25

**Authors:** Lewis Hsu

**Affiliations:** 1 University of Illinois at Chicago, Chicago, Illinois, USA

This Policy Brief presents the benefits of hydroxyurea (HU) for sickle cell disease (SCD) and urges policy changes to increase access to hydroxyurea in Tanzania. 

 Current data about SCD are concisely presented, including the large number of Tanzanians affected, the high morbidity and mortality, and the range of treatments available. The solid evidence on safety and benefits of HU for reducing SCD acute and chronic complications are presented, as well as ease of use. The policy steps proposed are very likely to increase access to HU, and very likely to reduce morbidity and mortality of SCD in Tanzania. 

 Minor critique

 Introduction, third paragraph. "HU prevents malaria infection" seems too strong. Consider modifying to be more precise: "HU reduces malaria mortality" (Olupot-Olupot P, Tomlinson G, Williams TN, Tshilolo L, Santos B, Smart LR, McElhinney K, Howard TA, Aygun B, Stuber SE, Lane A, Latham TS, Ware RE. Hydroxyurea treatment is associated with lower malaria incidence in children with sickle cell anemia in sub-Saharan Africa. Blood. 2023 Mar 23;141(12):1402-1410. doi: 10.1182/blood.2022017051.) 

 There is strong evidence that blood transfusion and hospitalizations are reduced by HU. Will the reduction per person be offset by the greater number of individuals with SCD surviving to adulthood on HU in Tanzania?

 Consider clarifying in this Policy Brief whether achieving the goal of 50% reduction of SCD-related deaths assumes that HU is started at a specific age (9 months? 2 years?) and a minimum proportion of the individuals with SCD?

 The paper focuses on increasing access to HU, which is necessary but not sufficient for large-scale implementation of HU for the SCD population in Tanzania. Will a public education campaign be needed to persuade parents that SCD could be less fatal with hydroxyurea, and accept the costs of coming to continue prescriptions for young children? What will be the effect on the patient volume of primary health centers? Can the hydroxyurea and lab test monitoring be tracked adequately in settings other than the tertiary care medical centers? Will patients stay consistently on the treatment, especially in the 5-17year-old age range which when previous work from Tanzania showed were likely to be "Lost to Follow-up" (LTFU)? *(Masamu U, Sangeda RZ, Kandonga D, Ondengo J, Ndobho F, Mmbando B, Nkya S, Msami K, Makani J. Patterns and patient factors associated with loss to follow-up in the Muhimbili sickle cell cohort, Tanzania. BMC Health Serv Res. 2020 Dec 14;20(1):1141. doi: 10.1186/s12913-020-05998-6. PMID: 33317526; PMCID: PMC7737273.)* **Could lessons be learned from implementation of ART drug use in HIV-infected patients, or even build SCD services with the same infrastructure?** Would systems and programs that lower LTFU in  HIV/AIDS be applied to SCD: adherence support programs, outreach to patients, and community-based adherence support clubs?

Does the paper provide a comprehensive overview of the policy and the context of its implementation in a way which is accessible to a general reader?

Yes

Is the discussion on the implications clearly and accurately presented and does it cite the current literature?

Yes

Are the recommendations made clear, balanced, and justified on the basis of the presented arguments?

Yes

Reviewer Expertise:

Sickle cell disease, implementation science, community engagement

I confirm that I have read this submission and believe that I have an appropriate level of expertise to confirm that it is of an acceptable scientific standard.

## References

[1] : Hydroxyurea treatment is associated with lower malaria incidence in children with sickle cell anemia in sub-Saharan Africa. *Blood* .2023;141(12) : 10.1182/blood.2022017051 1402-1410 10.1182/blood.2022017051 36375125PMC10273078

[2] : Patterns and patient factors associated with loss to follow-up in the Muhimbili sickle cell cohort, Tanzania. *BMC Health Serv Res* .2020;20(1) : 10.1186/s12913-020-05998-6 1141 10.1186/s12913-020-05998-6 33317526PMC7737273

[3] : Strategies to improve antiretroviral therapy (ART) initiation and early engagement among men in sub-Saharan Africa: A scoping review of interventions in the era of universal treatment. *Trop Med Int Health* .2023;28(6) : 10.1111/tmi.13880 454-465 10.1111/tmi.13880 37132119PMC10354296

[4] : Interventions to improve early retention of patients in antiretroviral therapy programmes in sub-Saharan Africa: A systematic review. *PLoS One* .2022;17(2) : 10.1371/journal.pone.0263663 e0263663 10.1371/journal.pone.0263663 35139118PMC8827476

# Peer review: Reviewer response for version 1

**DOI:** 10.5256/f1000research.123006.r193815

**Published:** 2023-08-16

**Authors:** Angela Rankine- Mullings

**Affiliations:** 1 The University of the West Indies, Kingston, Jamaica

The paper summarizes reasons for underutilization of hydroxyurea in Tanzania among persons with sickle cell disease and gives recommendation on how to promote access of hydroxyurea to persons with sickle cell disease. Importantly the authors show how existing resources can be utilized to improve accessibility of hydroxyurea in Tanzania. I believe this is an extremely important topic and this manuscript is very helpful to inform public health interventions aimed at improving disease outcome among persons with sickle cell disease. Thus, I reviewed this paper with great appreciation of the authors efforts.

 Please note minor corrections and suggestions:

1. The term Persons/individuals with SCD is preferred to SCD individuals.

Abstract: The writers may wish to add to the phrase “clinicians with a minimum of a Bachelor of Medicine to prescribe HU” **after appropriate basic training to prescribe hydroxyurea for persons with sickle cell disease.**

 This is not critical, and I leave this to the author to decide, Based on my experience optimizing hydroxyurea treatment among persons with sickle cell disease is also dependent on physician knowledge and experience.

 Figure 1 should be referenced and labelled comprehensively to stand alone and represent the manuscript fully if anyone should read the figure without reading the text. For example:

 Figure 1. Summary of the reasons found in previous qualitative research in the underutilization of HU among individuals with SCD in Tanzania and its consequences ^6, 7^ 

 Actions to improve utilization of HU among individuals with SCD in Tanzania should be categorized for easier reading. For example:

**Recommendations to improve the availability of hydroxyurea among persons with sickle cell disease in Tanzania**

- 6. Providing a more supportive environment to local pharmaceutical manufacturers in terms of more subsidization of raw materials and infrastructures for manufacturing of HU for SCD in Tanzania **(in collaboration with Ministries responsible for Finance, Industry, and Business)**.
  • This will help to realize priory number VIII in the health sector strategic plan 2021-2026 ^12^ which aims at “ *Improvement of research and development in health services to establish and strengthen research mechanisms on domestic pharmaceutical manufacturing that meet international standards for domestic and export use*”.
- 7. Creating (in collaboration with health research institutions) an easily accessible platform of reliable data on the burden of SCD and the need for HU in Tanzania which will help local pharmaceutical importers and manufacturers during the establishment of estimated demand and application for registration of HU.

**Recommendations to increase the accessibility of hydroxyurea to persons with SCD in Tanzania **

- 1. Extending prescription of HU to patients with SCD attending regional and district hospitals which have laboratory facilities for monitoring of blood parameters for patients on treatment.
- 3. Discussing with NHIF and remove the need for a special permit when issuing HU to SCD individuals.

**Recommendations to improve patient acceptability and affordability of hydroxyurea to persons with SCD in Tanzania**

- 2. Re-categorizing HU as the medication for cancer and non-cancer diseases.
- 4. Providing HU to individuals with SCD under a vertical program.
  • Capitalize on the experiences from tuberculosis, HIV/AIDS, and Neglected Tropical Diseases control programs.
- 5. Including HU in the subsidization scheme as an additional incentive on top of its inclusion in the Tanzania Orphan Drug Regulation of 2018.
  - Capitalize on the experiences from antimalarial medications, particularly Artemether-Lumefantrine.

It would be interesting to examine the experience of hydroxyurea accessibility in Tanzania among patients that are prescribed hydroxyurea for other indications outside of sickle cell disease. Is it easily available or are there issues with supply and demand? Networking with health care providers in other areas where hydroxyurea is prescribed may also be useful when setting up the “accessible platform of reliable data on the burden of SCD and the need for HU in Tanzania”

Does the paper provide a comprehensive overview of the policy and the context of its implementation in a way which is accessible to a general reader?

Yes

Is the discussion on the implications clearly and accurately presented and does it cite the current literature?

Yes

Are the recommendations made clear, balanced, and justified on the basis of the presented arguments?

Yes

Reviewer Expertise:

Sickle Cell Disease and Hydroxyurea Utilization for Improved Disease Outcomes

I confirm that I have read this submission and believe that I have an appropriate level of expertise to confirm that it is of an acceptable scientific standard.
